# Targeting immune cell migration as therapy for inflammatory disease: a review

**DOI:** 10.3389/fimmu.2025.1650760

**Published:** 2025-09-25

**Authors:** Bingfeng Song, Wenbo Guo, Ying He, Xingli Yao, Jintang Sun, Shijun Wang

**Affiliations:** ^1^ College of Traditional Chinese Medicine, Shandong University of Traditional Chinese Medicine, Jinan, China; ^2^ Research Center for Basic Medical Sciences, Qilu Hospital of Shandong University, Jinan, China; ^3^ Department of Rehabilitation, Shandong Provincial Hospital of Traditional Chinese Medicine, Jinan, China; ^4^ Pediatric Outpatient Department of Shandong Provincial Hospital, Jinan, China

**Keywords:** immune cells, migration, inflammatory diseases, chemokines, integrins, targeted therapy

## Abstract

Immune cell migration plays a pivotal role in coordinating inflammatory responses and maintaining immune surveillance. Here, we provide a comprehensive overview of the migratory behaviors of key immune cell subsets, including Th1, Th2, regulatory T cells, macrophages, dendritic cells, and neutrophils and the molecular mechanisms that guide their trafficking from lymphoid organs to inflamed tissues. We highlight the stepwise migration cascade: priming in secondary lymphoid organs, trafficking through blood vessels, adhesion to endothelium, and extravasation into tissues. Each step is critically regulated by chemokines, selectins, integrins, and proteases. We also examine current pharmacological strategies that target immune cell migration in inflammatory diseases, such as integrin blockers and chemokine receptor antagonists, emphasizing both therapeutic potential and clinical limitations. In addition, we discuss emerging technologies including intravital imaging, CRISPR-based screening, and computational modeling that provide novel insights into immune cell dynamics and may guide the development of next generation migration-targeted therapies. Overall, this review integrates fundamental immunological principles with translational medicine by identifying key challenges, unresolved controversies, and future directions in the therapeutic modulation of immune cell migration.

## 1 Introduction

The inflammatory response is a complex biological process characterized by the migration of leukocytes from the bloodstream into affected tissues, such as the visceral mucosa. Springer and Ley et al. demonstrated that this process involves not only the circulation of lymphocytes but also the active trafficking of antigen-presenting cells, including dendritic cells (DCs) and macrophages, from peripheral tissues to secondary lymphoid organs where T cell priming occurs (1, 2). This step is critical for initiating adaptive immunity and shaping effector cell responses, as emphasized by Banchereau and Steinman, and Belkaid and Hand (3, 4). Consequently, targeting immune cell migration has emerged as a central therapeutic strategy in inflammatory diseases.

Notably, inflammatory diseases demonstrate distinct organ/tissue targeting, with manifestations ranging from systemic to localized patterns. Systemic disorders such as systemic lupus erythematosus affect multiple organs (e.g., skin, lungs, kidneys, vasculature, and CNS) due to widespread immune dysregulation (5–7). By contrast, localized conditions including psoriasis, inflammatory bowel diseases (ulcerative colitis, Crohn’s disease), multiple sclerosis, and Alzheimer’s disease primarily target specific tissues (8–12). These observations underscore the need to investigate immune mechanisms across both systemic and localized contexts to guide targeted therapies.

**Figure 1:**
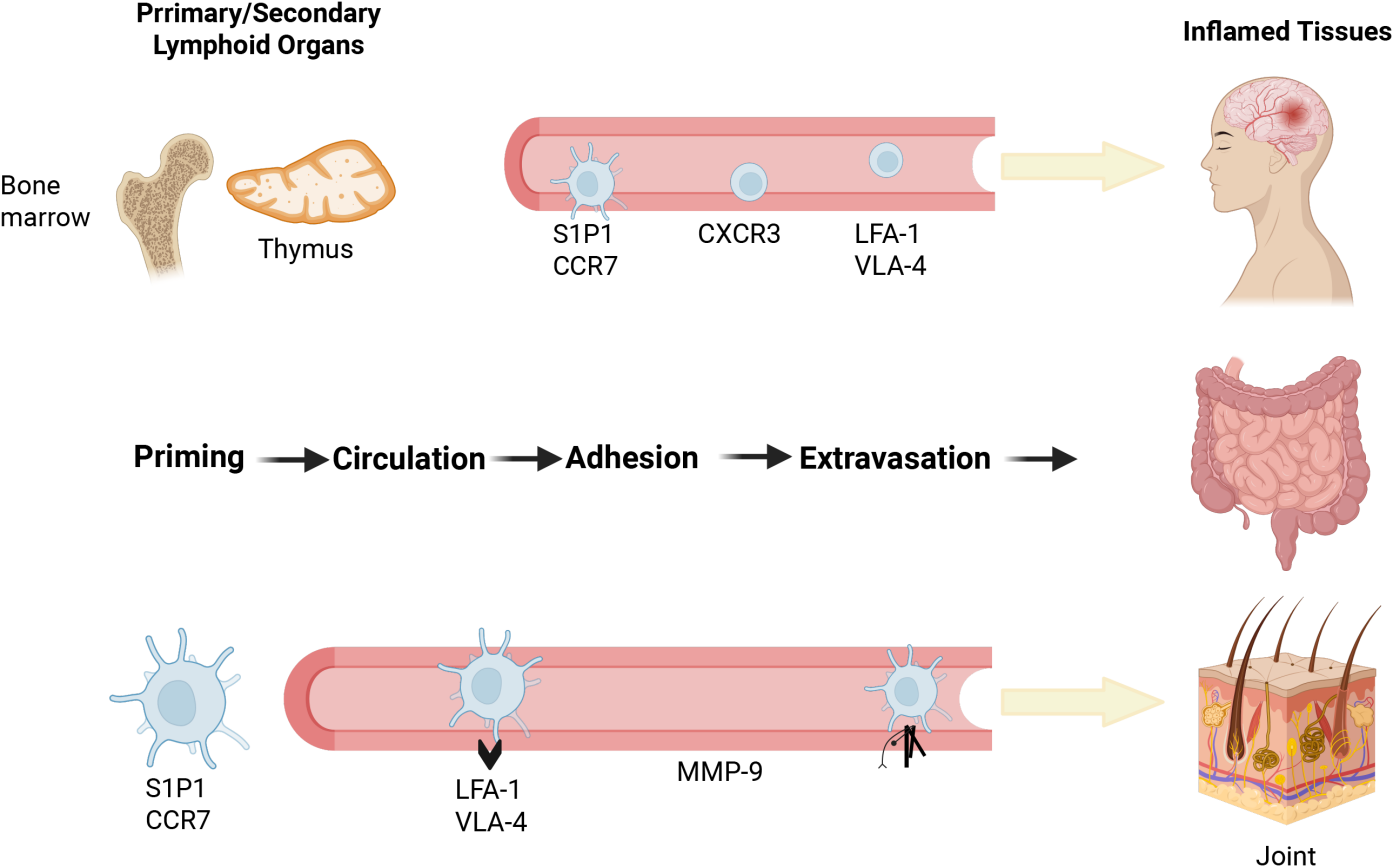
The stepwise process of immune cell migration during inflammation. Schematic illustration of leukocyte trafficking from primary and secondary lymphoid organs to inflamed peripheral tissues. The migration involves four main stages: priming in lymphoid tissues, circulation in blood, adhesion to vascular endothelium, and extravasation into inflamed sites. Key regulators include chemokines (e.g., S1P1, CCR7), integrins (LFA-1, VLA-4), and matrix metalloproteinases (e.g., MMP-9).

A hallmark of inflammatory diseases is the infiltration of functionally distinct inflammatory cell subsets. Environmental signals activate effector cell subsets to secrete or express migration-related molecules including cytokines and chemokines, enabling tissue-specific homing. Understanding the recruitment mechanisms of these subsets is therefore critical for developing therapies that prevent pathological accumulation of effector cells. A promising approach involves designing agents that selectively inhibit inflammatory cell trafficking and aggregation while preserving protective tissue-resident immunity (13, 14). Such targeted strategies minimize pathological infiltration without compromising host defense or tissue homeostasis (**Figure 1**).

This review comprehensively addresses the molecular mechanisms underlying leukocyte subset migration to organs, their roles in inflammatory disease pathogenesis, and the therapeutic strategies to inhibit this process. By integrating current advances in this field, this work not only summarizes established approaches but also emphasizes innovative therapies designed to enhance specificity while minimizing adverse effects. Unlike previous methodologies that were limited to single immune cell types or isolated pathways, such as the three-dimensional tracking of T cell migration in the intestinal mucosa described in Current Protocols, the present study establishes a multi-level integrative framework that connects molecular mediators, cellular dynamics, and organ-specific pathophysiology to translational applications. We systematically compare migration patterns across diverse immune cell subsets (T cells, B cells, macrophages, dendritic cells, mast cells, and granulocytes) and characterize their interactions with chemokines, adhesion molecules, and proteases. Furthermore, we bridge fundamental mechanistic insights with clinical relevance by summarizing both approved drugs and emerging technologies, including intravital imaging, CRISPR-based screening, and smart drug-delivery platforms. Collectively, these elements highlight what has been achieved here: a unifying framework for precision targeting of immune cell migration that advances safer and more effective anti-inflammatory therapies.

Nevertheless, significant challenges remain in translating preclinical insights into effective clinical interventions, particularly in balancing immune suppression with host defense. Addressing these gaps will be crucial for developing next-generation strategies with durable therapeutic benefits.

## 2 Immune cell subpopulations involved in inflammation

### 2.1 Lymphocytes

#### 2.1.1 Initial migration and T-cell priming

Newly generated lymphocytes emerge from primary lymphoid organs (bone marrow for B cells, thymus for T cells) into the bloodstream and subsequently migrate, a process precisely guided by distinct patterns of surface protein expression. Nolz and Wang et al. reported that the expression profiles and functional states of these migration-regulating proteins are dynamically modulated by inflammatory signals, resulting in profound changes in lymphocyte trafficking and effector functions (15, 16).

Following maturation in primary lymphoid organs, lymphocytes enter systemic circulation and home to secondary lymphoid organs, principally lymph nodes and spleen where immune responses are initiated. Within these organs, lymphocytes interact with antigen-presenting cells (APCs), particularly dendritic cells and macrophages, to initiate T-cell priming (17, 18). Förster et al. showed that naïve T cells express CCR7, which binds to CCL19 and CCL21 secreted by dendritic cells, thereby directing T-cell migration into lymph nodes through high endothelial venules (18). Linsley et al. demonstrated that CD28 on T cells binds to CD80 or CD86 on APCs, delivering a costimulatory signal that stabilizes IL-2 transcription, activates MAPK and NF-κB signaling pathways, and promotes clonal expansion of CD4^+^ and CD8^+^ T cells (19). Notably, while CD80 exhibits higher affinity for both CD28 and CTLA-4, CD86 plays a more prominent role in supporting Treg homeostasis and may be less susceptible to CTLA-4-mediated inhibition (20). Additionally, PD-L1 can bind CD80 in cis on the same APC surface. This interaction enhances CD28-mediated co-stimulation while simultaneously attenuating PD-1 inhibitory signaling (21). T-cell receptor (TCR) recognition of peptide-MHC complexes ensures antigen specificity, while integrins such as LFA-1 (on T cells) binding to ICAM-1 (on APCs) stabilize the immune synapse (22). Together, these receptor-ligand interactions provide the molecular basis for effective T-cell priming by coordinating trafficking, co-stimulation, antigen recognition, and immune synapse stabilization. Importantly, dissecting these pathways not only deepens our mechanistic understanding but also highlights potential therapeutic targets for enhancing immunotherapy efficacy and promoting tolerance in autoimmune or transplant settings.

#### 2.1.2 Activated T-cell trafficking to inflammatory sites

Following activation, T cells exit secondary lymphoid organs, re-enter systemic circulation, and migrate to inflammatory sites. During this process, they undergo extravasation mediated by chemokines, selectins, and integrins secreted by endothelial cells. Importantly, their trafficking is tightly regulated by homing receptors such as CCR7, CXCR3, and α4β7 integrin, which respond dynamically to local inflammatory cues (23, 24). Targeting these mechanisms provides precision strategies to modulate pathological immune cell trafficking.

#### 2.1.3 Effector subsets, therapeutic implications, and clinical challenges

Significant progress has been made in elucidating the links between T-cell migration and inflammation. Giovenzana et al. summarized that blocking pathogenic T-cell trafficking has shown therapeutic benefits across diverse inflammatory diseases including type 1 diabetes, multiple sclerosis, Crohn’s disease, rheumatoid arthritis, atherosclerosis, and psoriasis (25)(**Table 1**). Rossi et al. further demonstrated that α4β7 integrin blockade impairs Th17 cell migration into the spinal cord leptomeninges in EAE, thereby attenuating neuroinflammation (26). Consequently, therapeutic strategies now focus on pharmacologically inhibiting Th1 migration and reestablishing Th1/Th2 balance (27–29). In contrast, Th2 cell infiltration, accompanied by elevated IL-4, IL-5, and IL-13 secretion, characterizes inflammation in parasitic infection and asthma (30, 31). Estrada Brull et al. highlighted the importance of Treg trafficking and their dynamic interplay with peripheral tissues in maintaining immune regulation (32).

**Table 1:** Migration features and pathological roles of lymphocyte subsets.

Subset	Key receptors/ligands (location)	Pathological relevance
Th1 cells	CXCR3, CCR5 (Th1)↔CXCL9/10/11, CCL3/4/5 (inflamed tissues, endothelium); LFA-1, VLA-4 (Th1) ↔ICAM-1, VCAM-1 (endothelium)	Chronic inflammation; type 1 diabetes, MS, Crohn’s disease, RA, atherosclerosis, psoriasis
Th2 cells	CCR3, CCR4, CCR8 (Th2) ↔ CCL11, CCL17, CCL1 (epithelium, DCs); α4β7, LFA-1 (Th2) ↔ MAdCAM-1, ICAM-1 (endothelium)	Airway inflammation, asthma, parasitic infections
B cells/plasma cells	CXCR5 (B cells) ↔ CXCL13 (FDCs); α4β1 (B cells) ↔ VCAM-1 (HEV/endothelium); CXCR3 (plasma cells) ↔ CXCL9/10/11 (inflamed tissues)	Autoantibody-mediated autoimmunity; SLE, allograft rejection
Treg cells	CCR4, CCR7 (Tregs) ↔ CCL17/22, CCL19/21 (DCs, HEVs); LFA-1, α4β7 (Tregs) ↔ ICAM-1, MAdCAM-1 (endothelium)	Immune tolerance; impaired trafficking leads to uncontrolled inflammation

Nevertheless, therapeutic interventions targeting inflammatory effector cell migration must consider the overlapping migratory mechanisms shared by regulatory T cells (Tregs). Tregs play a crucial role in maintaining immune homeostasis, and any disruption to their recruitment could inadvertently compromise immune regulation. Consequently, while inhibitors that block the migration of inflammatory effector cells hold promise, they may also influence Tregs recruitment, presenting challenges in balancing efficacy with safety. Current studies are intensely focused on understanding the pathological effects of such therapies, including the potential increased susceptibility to infections (13, 33), with the ultimate goal of developing refined treatment strategies that maintain protective immunity while achieving disease control. Future work must address how to selectively modulate pathogenic effector cell trafficking without impairing Treg-mediated tolerance. Clinically, achieving this balance will be critical for developing next-generation therapies that are both effective and safe.

### 2.2 Monocytes and macrophages

#### 2.2.1 Macrophages

Monocytes play an essential immune function by differentiating into macrophages and DCs, both of which are key players in the pathological processes of various inflammatory diseases (34–36). Activated macrophages are central participants in Th1-mediated diseases such as multiple sclerosis, arteriosclerosis, and rheumatoid arthritis (37, 38). Serbina et al. demonstrated that macrophage trafficking is strongly regulated by the CCR2-CCL2 axis, which promotes their accumulation at inflammatory sites and sustains disease progression (39). These macrophages exacerbate local immune responses through the secretion of pro-inflammatory cytokines such as TNF-α, IL-1β, IL-6, IL-12, IL-23, IL-17, and IL-18, and the generation of reactive oxygen species, thereby driving tissue remodeling and sustaining chronic inflammatory states (**Table 2**). The critical role of macrophages in these processes highlights their transport proteins as promising therapeutic targets for chronic inflammatory diseases. Strategic modulation of macrophage migration and effector functions may allow inflammation control while preserving their indispensable roles in host defense and tissue repair.

**Table 2:** Macrophage-derived pro-inflammatory cytokines.

Cytokine	Primary roles & impact in chronic inflammation
TNF-α	Triggers immune activation, mediates cell death, and amplifies inflammation
IL-1β	Promotes inflammatory signaling and pain, and is upregulated in injured or infected tissues
IL-6	Drives acute-phase response and systemic inflammation, and supports immune cell differentiation
IL-12	Stimulates Th1 responses and IFN-γ production
IL-23	Promotes Th17 differentiation and IL-17 secretion
IL-17	Enhances chemokine production, recruits neutrophils, and contributes to tissue remodeling and angiogenesis
IL-18	Induces IFN-γ in immune cells, and participates in autoimmune and neuroinflammatory processes

#### 2.2.2 Dendritic cells

DCs represent a specialized class of APCs that functionally interconnect the innate and adaptive immune systems. Heras-Murillo et al. demonstrated that conventional type-1 DCs (cDC1s) can induce durable immune memory and prevent tumor relapse, underscoring the therapeutic potential of DC-based interventions (40). Jiménez-Cortegana et al. further emphasized the dualistic nature of DCs, acting as both promoters and regulators of immune responses in different disease contexts, thereby highlighting their yin–yang role in inflammatory pathogenesis (41). These cells regulate immune responses by activating CD4^+^ and CD8^+^ T cells, and undergo substantial phenotypic and functional maturation during migration to target tissues. Current studies have delineated specialized subsets, particularly conventional DCs (cDCs) and plasmacytoid DCs (pDCs), which contribute differently to pathogenesis and immune regulation (40, 42). cDCs can be subdivided into cDC1 and cDC2, with distinct antigen-presenting and T-cell priming capacities. cDC1s specialize in cross-presentation and CD8^+^ T cell activation, whereas cDC2s preferentially prime CD4^+^ T cells and promote their differentiation into Th2 or Th17 lineages under local cytokine cues (43, 44). Transcriptomic and proteomic profiling has shown that cDC2s express an expanded repertoire of MHC class II-linked co-stimulatory molecules, enhancing their ability to present exogenous antigens to naïve CD4^+^ T cells. Functionally, this enables pathogen-tailored responses, promoting Th1 polarization during viral infections while driving Th17 responses against fungal invasion. Deciphering these distinctions is crucial for the design of DC-targeted vaccines and immunotherapies (45, 46).

DCs subsets are highly heterogeneous, differing in function, cytokine production, and tissue distribution, which underpins their distinct contributions to immune regulation in health and disease. In psoriasis, cDC2s promote IL-23-dependent Th17 polarization, driving epidermal inflammation and keratinocyte hyperproliferation (47). In systemic lupus erythematosus (SLE), pDCs act as major producers of type I interferons, which potently amplify autoimmune cascades (42). Therapeutic blockade of type I interferon signaling has shown encouraging results in preclinical studies and early clinical trials for SLE (48). Rodrigues et al. demonstrated that RORγt^+^ dendritic cells are required for the induction of peripheral regulatory T cells in response to oral antigens, and disruption of this subset can impair immune tolerance to gut microbiota, thereby driving pathogenic responses in inflammatory bowel disease (IBD) (49). Cabezón and Benítez-Ribas reported that while aberrant mucosal cDCs promote inflammatory T-cell activation, tolerogenic dendritic cells help maintain intestinal homeostasis, offering a therapeutic window for selective modulation (50).

#### 2.2.3 Therapeutic implications

Emerging strategies to modulate DC function are under active clinical investigation. Current approaches include TLR agonists as adjuvants in cancer vaccines, enhancing DC-mediated antigen presentation (51), cytokine inhibitors targeting IL-12/23 signaling to suppress DC-driven inflammation in immune-mediated diseases (52), and nanoparticle-based platforms for targeted delivery to DC subsets (53). These technologies aim to harness the intrinsic antigen-presenting properties of DCs while minimizing systemic immune activation.

In conclusion, targeting the migratory and functional dynamics of macrophages and DCs offers a promising direction for anti-inflammatory therapies. A deeper understanding of the molecular mechanisms regulating their trafficking and activation enables the development of innovative treatments that target the underlying pathophysiology of inflammation while preserving systemic immune competence. Clinically, the challenge remains to selectively inhibit pathogenic macrophage and DC subsets without impairing protective immunity. Future research should focus on identifying subset-specific markers and therapeutic windows that allow precise intervention with minimal adverse effects.

### 2.3 Mast cells and granulocytes

#### 2.3.1 Mast cells

Bone marrow-derived mast cells are primarily located around arterioles and venules in peripheral tissues, where they maintain tissue homeostasis and mediate inflammatory responses. During inflammation, pro-inflammatory signals activate mast cells, triggering degranulation and the release of histamine, TNF-α, and cytokines such as IL-4, IL-5, IL-13, and IL-17A. This cascade increases postcapillary venule permeability and promotes targeted immune cell recruitment, thereby amplifying allergic inflammation. It has been reported that Th2 and Th17 cells further exacerbate asthma by driving eosinophil accumulation, IgE synthesis, and airway remodeling (31, 54). Targeting these cytokine-dependent interactions provides therapeutic opportunities. For instance, inhibition of mast cell-derived TNF-α and histamine reduces neutrophil and eosinophil infiltration in models of allergic asthma and colitis (55, 56). Pharmacological agents such as cromolyn sodium and the tyrosine kinase inhibitor masitinib effectively block mast cell activation and leukocyte recruitment, thereby reducing tissue inflammation and providing symptom relief (57, 58). These findings highlight mast cell-targeted inhibition of recruitment signaling as a viable therapeutic approach for chronic inflammatory disorders.

#### 2.3.2 Granulocytes

Granulocytes, including neutrophils, eosinophils, and basophils, are critical components of innate immunity. Neutrophils, the most abundant subset, rapidly accumulate at acute inflammatory sites within hours and extravasate into tissues through interactions between adhesion molecules and endothelial receptors. This response plays essential roles in host defense but also contributes to ischemia-reperfusion injury and severe conditions such as myocardial infarction, stroke, shock, and acute respiratory distress syndrome (ARDS). Sawant et al. demonstrated that mast cell recruitment into the tumor microenvironment is modulated by CXCL6-CXCR2 signaling (59). Abonia et al. demonstrated that mast cell progenitors’ homing to the intestine depends on CXCR2 expression (60). These findings highlight the broader translational relevance of CXCR2 signaling in granulocyte-targeted interventions. Granulocyte migration is orchestrated by chemokine, cytokine, and lipid mediator gradients. Among these, lipid mediators such as leukotrienes and specialized pro-resolving mediators (SPMs) regulate neutrophil migration, activation, and clearance, thereby coordinating both the initiation and resolution of inflammation (61). Therapeutic approaches under investigation include CXCR2 antagonists (e.g., navarixin and danirixin), anti-integrin or anti-selectin antibodies (2), and approaches targeting neutrophil extracellular trap (NET) formation. Clinically, CXCR2 inhibitors have reduced neutrophilic inflammation in COPD and asthma (62, 63), while CXCL8-targeting agents (e.g., reparixin) show potential in pancreatitis and transplant rejection (64).

#### 2.3.3 Therapeutic implications

By strategically targeting mast cells and granulocytes, researchers are advancing next-generation anti-inflammatory therapies aimed at achieving precise immunomodulation. This approach seeks to suppress pathological inflammation while preserving host defense mechanisms, thereby addressing the limitations of conventional broad-spectrum immunosuppression. Clinically, the challenge lies in distinguishing pathogenic from protective responses, as excessive suppression may increase infection risk. Future studies should focus on identifying subset-specific markers and therapeutic windows that allow selective inhibition of harmful mast cell and granulocyte activity without compromising protective immunity. A major research gap remains the lack of reliable biomarkers to differentiate pathogenic from homeostatic responses, which represents a critical barrier to precision therapy (**Table 3**).

**Table 3:** Migration features and pathological relevance of mast cells and granulocytes.

Subset	Migration features/key molecules	Pathological relevance & diseases
Mast cells	Perivascular; activated by inflammation; release histamine, TNF-α, IL-4/5/13/17A; recruit eosinophils/neutrophils	Amplify allergic inflammation; asthma, colitis, chronic allergy
Neutrophils	CXCR1/2; extravasation via selectins/integrins; recruited by CXCL8, leukotrienes, SPMs	Acute inflammation; ischemia–reperfusion injury, COPD, asthma, ARDS, MI, stroke
Eosinophils/Basophils	CCR3–eotaxin axis; activated by IL-4, IL-5, IL-13; IgE-mediated responses	Allergic inflammation, asthma, parasitic infections

## 3 Key molecular regulators of immune cell migration

**Figure 2:**
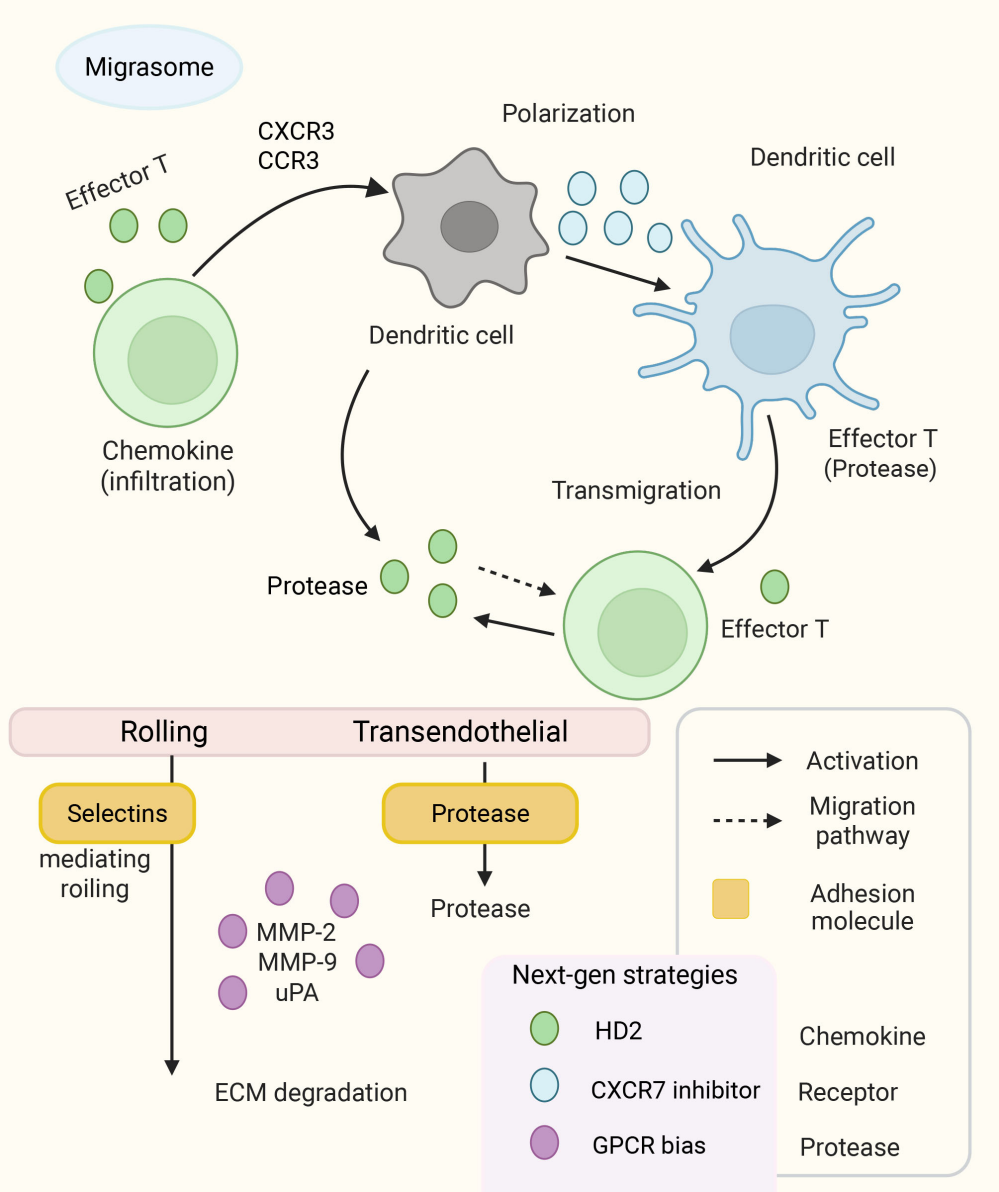
Key molecular regulators and next-generation strategies of immune cell migration in inflammation. Immune cell migration is a multistep cascade involving chemokines, adhesion molecules, and proteases. Circulating effector T cells and dendritic cells are sequentially activated by chemokine-receptor interactions (e.g., CXCR3/CCR3, CCR7), undergo polarization, and extravasate via transendothelial migration. Adhesion molecules such as selectins mediate rolling, while proteases (e.g., MMP-2, MMP-9, uPA) degrade the extracellular matrix (ECM) to facilitate transmigration. The arrows represent distinct processes: solid arrows indicate cellular activation, while dashed arrows indicate migration pathways. The colored balls denote different classes of molecules: green for chemokines, blue for receptors, yellow boxes for adhesion molecules, and purple for proteases. The lower panel highlights emerging therapeutic approaches, including the pan-chemokine inhibitor HD2 (green), CXCR7 inhibitors (blue), and biased GPCR modulators (purple), which collectively represent next-generation strategies to modulate pathological immune cell trafficking.

Immune cell migration is a tightly regulated multistep process essential for immune surveillance and inflammatory responses (65). In addition to these classical pathways, emerging mechanisms such as migrasome-mediated cytokine delivery have also been implicated (66). The cascade begins with circulating immune cells adhering to vascular endothelium via adhesion molecules and chemokines, followed by cytoskeletal polarization for extravasation. After traversing the endothelial barrier, cells migrate through stromal compartments and extracellular matrix (ECM) along chemotactic gradients to reach inflammatory sites. This coordinated movement is governed by chemokine–receptor interactions, adhesion molecules, and proteases (67). Such a precisely regulated molecular network ensures efficient and timely immune cell trafficking, thereby enabling effective immune responses against infections and tissue injury. Importantly, elucidating these migratory mechanisms provides valuable insight into novel therapeutic targets for immune-mediated diseases (**Figure 2**).

### 3.1 Chemokines and chemokine receptors

Recent breakthroughs have substantially advanced our understanding of chemokine-receptor networks in immune cell migration. Kryukova et al. identified a 16-mer peptide “HD2” via phage display that simultaneously binds and inhibits CCL/CXCL chemokines, serving as a blueprint for pan-chemokine inhibitor design (68). In parallel, quantitative structure-activity relationship (QSAR) analysis and molecular docking have facilitated the discovery of novel CXCR7 inhibitors, enabling precise targeting of tumor angiogenesis and metastasis (69). Additionally, biased signaling in G-protein-coupled receptor (GPCR) transduction has emerged as a critical concept: by designing small molecules that selectively activate β-arrestin while sparing G protein pathways, more effective and safer therapeutics can be developed (70).

Chemokines, a specialized class of cytokine polypeptides, critically regulate immune cell migration through GPCR activation (71, 72). Classical chemokine-receptor pairs such as CXCL10/CXCR3, CCL2/CCR2, and CCL5/CCR5 are well-characterized under both physiological and inflammatory conditions (73). During inflammation, chemokines enhance leukocyte adhesion to vascular endothelium and promote endothelial gap formation, facilitating immune cell extravasation. Their rapid action and subset specificity allow precise spatiotemporal control of leukocyte trafficking (74, 75). Chemokine receptors on leukocyte surfaces serve as “migratory passports”, guiding specific cell subpopulations through tissue barrier checkpoints (75).

The CCL19/21–CCR7 axis exemplifies this mechanism: during antigen exposure or vaccination, CCR7-mediated chemotaxis directs dendritic cells (DCs) from peripheral tissues to lymph nodes, thereby initiating T cell responses (76, 77). Recent studies show that CCL21 provides a spatial gradient for DC lymphatic entry, whereas CCL19 establishes self-generated chemokine gradients via CCR7 internalization, synchronizing DC–T cell interactions (77). Moreover, boosting CCL19/21 signaling (e.g., with adjuvants or focused ultrasound) enhances DC vaccine efficacy by improving lymph node homing and antitumor immunity (78, 79). Zhang et al. reviewed chemokine-GPCR pathways and their modulatory roles in inflammation (80). Hu et al. summarized the role of cell adhesion molecules in fibrotic diseases, suggesting that targeting adhesion pathways may complement chemokine inhibition in inflammatory settings (81). These findings underscore the clinical potential of chemokine-targeted strategies in cancer, vaccination, and inflammatory disorders. However, challenges such as pathway redundancy, compensatory mechanisms, and safety concerns highlight the need for more selective and context-specific therapeutic approaches. Future efforts should prioritize developing next-generation chemokine modulators that achieve precise immune control while minimizing systemic toxicity.

### 3.2 Cell adhesion molecules

Cell adhesion molecules (CAMs), particularly selectins, integrins, and mucin-associated hyaluronate receptors, mediate essential cell-cell and extracellular matrix (ECM) interactions that critically regulate immune cell trafficking during inflammatory responses (81). These molecules provide precise spatiotemporal control of leukocyte adhesion and migration, forming the molecular basis for targeted immune surveillance.

L-selectin, expressed predominantly by circulating immune cells, binds vascular sulfated sialoglycoproteins to initiate leukocyte rolling adhesion. While indispensable for tissue repair and immune surveillance, dysregulated L-selectin activity contributes to pathological inflammation and tissue damage in infectious and inflammatory diseases (82).

Integrins, a family of heterodimeric proteins composed of α and β subunits, represent another critical class of CAMs governing immune cell migration. The β1 integrin subfamily, including VLA-1, VLA-2, VLA-4, VLA-5, and VLA-6, regulates leukocyte passage across the vascular basement membrane, through the ECM, and into stromal and endothelial layers. By mediating adhesion and spreading on ECM proteins, these integrins enable tissue infiltration during inflammatory responses (83).

In addition, L-selectin, P-selectin glycoprotein ligand-1 (PSGL-1), and α4 integrin cooperate to mediate immune cell adhesion to vascular endothelial cells (13). Initially, L-selectin and P-selectin promote rolling interactions that slow leukocytes and allow transient attachment, a prerequisite for sensing chemotactic cues from inflamed tissues. From a translational perspective, α4-integrin blockade has emerged as an effective therapeutic strategy in multiple sclerosis: natalizumab, a monoclonal antibody against α4-integrin, significantly reduces relapse rates and delays disability progression (84). Vedolizumab, a selective α4β7 integrin inhibitor, has demonstrated efficacy in ulcerative colitis and Crohn’s disease and is FDA-approved for these indications (85, 86), underscoring the therapeutic relevance of α4-integrin biology.

Mechanistically, E-selectin and α4 integrins further decelerate rolling leukocytes, enabling chemokines to rapidly activate β2 and/or α4 integrins on the immune cell surface. This activation induces firm adhesion to the endothelium, a prerequisite for transendothelial migration (87). Ultimately, immune cells extravasate into the surrounding tissue, orchestrating the inflammatory response. However, dysregulated CAM activity can lead to excessive infiltration and drive the pathogenesis of various inflammatory disorders. Thus, elucidating the molecular mechanisms underlying selectins, integrins, and related CAMs is critical for developing targeted strategies to modulate immune cell migration (13).

In summary, CAMs not only underpin the molecular logic of immune surveillance and inflammation but also represent actionable therapeutic targets in autoimmune and inflammatory diseases. Nevertheless, context-specific regulation of CAMs remains insufficiently understood, representing a key research gap for advancing their clinical translation.

### 3.3 Proteases

Functioning as proteolytic enzymes, proteases critically regulate immune responses through protein cleavage and modification (88, 89). However, current descriptions often fail to distinguish between the proteases mediating neutrophil, monocyte, and T cell migration. For example, MMP-9 plays a central role in neutrophil extravasation (90, 91), whereas MMP-2 is primarily associated with T cell infiltration. Clarifying such distinctions would enhance both mechanistic clarity and therapeutic relevance.

In the context of immune cell migration, proteases degrade extracellular matrix (ECM) components, enabling cellular transit through tissues to inflammatory or infected sites. These proteases comprise both secretory and cell-surface-associated subtypes. The most well-studied secretory proteases are matrix metalloproteins (MMPs) and urokinase-type plasminogen activator (uPA). MMPs cleave key ECM proteins, including collagen, elastin, and fibronectin, thereby facilitating immune cell infiltration (92). uPA promotes migration by converting plasminogen to plasmin, which degrades fibrin and other ECM components (93). Cell-surface proteases, expressed on endothelial and immune cells, serve dual functions as both ectoenzymes and adhesion receptors, regulating cell-ECM interactions through substrate cleavage and soluble factor generation. While proteases facilitate immune cell migration, their pleiotropic nature complicates therapeutic targeting. Given their diverse biological roles, protease inhibition risks off-target effects that may compromise immune function or tissue homeostasis. For example, inhibiting MMPs could potentially disrupt wound healing, tissue remodeling (94), or even the recruitment of non-inflammatory cells necessary for tissue repair. These challenges highlight the need for more targeted therapeutic strategies. A promising therapeutic approach involves developing tissue-specific drug delivery systems, such as antibody-conjugated nanoparticles that target inflamed endothelium, thereby minimizing systemic effects (95).

Notably, certain immune cells, such as lymphocytes and monocytes, can migrate independently of protease activity by physically deforming to traverse ECM spaces without enzymatic degradation (96). This protease-independent mechanism suggests that some immune cells rely on mechanical properties and interactions with other cellular structures, such as the actin cytoskeleton, to facilitate their movement. This finding underscores the complexity of leukocyte migration and highlights the importance of considering both enzymatic and mechanical mechanisms in therapeutic design.

Overall, although proteases are crucial regulators of immune cell migration, their varying functions across different immune cell types and tissue contexts highlight the complexity of this process. Future research should prioritize developing highly specific protease inhibitors or alternative strategies that selectively target pathological migration while minimizing side effects. However, despite promising preclinical evidence, several clinical trials targeting MMPs have failed. For instance, Sparano et al. conducted a randomized phase III trial of marimastat versus placebo in metastatic breast cancer and found no improvement in overall survival or PFS, along with significantly higher rates of musculoskeletal toxicity (63% vs 22%) (97). Similarly, Bissett et al. reported that the MMP inhibitor prinomastat failed to improve outcomes in advanced non-small-cell lung cancer, and was associated with frequent joint-related adverse events, leading to premature trial termination (98). Although these trials primarily investigated cancer rather than inflammatory diseases, their outcomes reveal fundamental challenges in targeting proteases therapeutically.

In summary, proteases are indispensable yet complex regulators of immune cell migration. Their cell type–specific and context-dependent roles underscore both their therapeutic potential and the difficulties in developing selective inhibitors. A deeper understanding of protease regulation in inflammatory versus homeostatic settings remains a major research gap, and addressing this will be crucial for advancing clinical translation.

## 4 Advances in anti-inflammatory drug targets based on migratory molecules

### 4.1 Therapeutic strategies targeting immune cell migration

The dual role of immune cell migration in both protective immunity and pathological inflammation has made it a compelling therapeutic target (99, 100). Current therapeutic strategies target various stages of the migratory cascade, utilizing both small-molecule inhibitors and biologic agents (101, 102). Sphingosine-1-phosphate (S1P) receptor modulators exemplify this approach. By binding S1P_1_ on lymphocytes, agents such as siponimod and ozanimod prevent lymph node egress, thereby reducing inflammatory infiltration into the CNS. Beyond immunomodulation, siponimod penetrates the blood-brain barrier and confers neuroprotective benefits, including remyelination and reduced gray matter atrophy. In phase III clinical trials, significant reductions in relapse rates and delayed disability progression have been demonstrated in multiple sclerosis (103–105).

Integrin inhibitors represent another clinically validated approach, with natalizumab serving as the prototype. This monoclonal antibody targets α4 integrins, blocking both VCAM-1 interactions in the CNS and MadCAM-1 binding in the gut. While effective in multiple sclerosis and Crohn’s disease, its association with progressive multifocal leukoencephalopathy highlights the risks of broad immune cell sequestration (106, 107). Vedolizumab, a selective α4β7 integrin inhibitor, has proven particularly effective in ulcerative colitis and Crohn’s disease. Phase III GEMINI I/II trials confirmed the therapeutic benefit of vedolizumab, leading to FDA approval for both UC and CD (108).

Emerging precision strategies aim to redirect beneficial immune subsets rather than broadly suppress immunity. Low-dose IL-2 selectively expands Tregs and has demonstrated clinical benefit in autoimmune conditions such as systemic lupus erythematosus (109). Engineered IL-2 muteins further improve specificity and pharmacokinetics, sustaining Treg expansion in preclinical models (110). In inflammatory bowel disease, low-dose IL-2 and cytokine-delivery nanoparticles have been shown to reduce colitis and restore mucosal tolerance (111). Nanoparticle-based approaches also enable chemokine modulation, such as CCL22 delivery promotes Treg recruitment and improves outcomes in experimental autoimmune encephalomyelitis (EAE) (112). Combination strategies targeting chemokine receptors have also been explored. For instance, CCR2-deficient mice are largely resistant to EAE, highlighting CCR2’s central role in monocyte recruitment to the CNS. Additionally, preclinical studies have investigated CCR1 and CCR2 blockade as therapeutic strategies to limit neuroinflammation in EAE and multiple sclerosis models (113). Collectively, these findings indicate that modulating immune cell trafficking provides therapeutic benefit while preserving immune surveillance. The field is moving toward precision strategies that separate pathological from protective migration.

Significant translational challenges remain, which can be divided into three principal barriers: compensatory migratory mechanisms that circumvent single-target strategies, the translational gap between preclinical findings and clinical application, and interspecies differences in receptor expression such as CCR5 and α4β7 (114, 115). Patients with elevated CCR5 expression, notably in HIV, multiple sclerosis, and IBD, may benefit from CCR5 antagonists such as maraviroc. In mice, oral maraviroc attenuated intestinal inflammation by reducing CCR5^+^ leukocyte recruitment, underscoring its therapeutic potential in IBD (116). Conversely, patients with predominant α4β7 integrin expression respond preferentially to vedolizumab. Accumulating evidence links α4β7^+^ lymphocyte abundance and transcriptional signatures of regulatory T cells to favorable treatment outcomes (117–119).

Bridging these receptor-specific insights into broadly effective therapies will require next-generation translational platforms, including humanized mouse models, functionally competent organoids, and organ-on-a-chip microfluidic systems (120–122). When combined with comprehensive multi-omics profiling, these technologies will enable systematic mapping of conserved migratory networks, strengthening target validation and supporting the rational design of precision immunotherapies. In addition to integrin antagonists and chemokine receptor inhibitors, S1P receptor modulators (e.g., fingolimod and siponimod) represent an important class of small-molecule therapies that restrict lymphocyte egress from lymph nodes, thereby limiting central nervous system infiltration in multiple sclerosis (105). Beyond blocking pathogenic cell entry, tolerance-inducing strategies are emerging, including low-dose IL-2, engineered IL-2 muteins, and CAR-Treg therapies can selectively expand Tregs, reduce inflammation, and provide long-term immune regulation (123–125).

In summary, therapies targeting immune cell migration have demonstrated clear efficacy in MS, IBD, and related inflammatory conditions. However, receptor redundancy, interspecies variability, and safety concerns underscore a key research gap: the urgent need for more selective, context-specific, and clinically translatable therapeutic platforms to fully realize the potential of migration-targeted immunotherapy.

### 4.2 Advanced *In Vitro* models for investigating immune cell chemotaxis

Recent breakthroughs in advanced *in vitro* systems, including organ-on-a-chip technology, 3D bioprinted tissues, and microfluidic platforms, have greatly improved the ability to mimic physiological microenvironments (126–128). These models provide precise control of chemokine gradients, tissue architecture, and cellular interactions, enabling detailed studies of immune cell chemotaxis under physiologically relevant conditions. Huh et al. and Bhatia et al. demonstrated that organ-on-a-chip devices, which employ microengineered channels lined with living cells, can replicate key structural and functional features of human organs while incorporating dynamic elements such as blood flow and mechanical stress (129, 130). These systems are particularly valuable for investigating immune cell migration across vascular and epithelial barriers. Recent studies, including those by Mazzaglia et al. and Cherukuri et al., demonstrated that 3D bioprinting techniques allow the spatial arrangement of diverse cell populations within extracellular matrices to construct immune-competent tissues, faithfully mimicking specialized niches such as lymphoid structures (131, 132). These platforms are indispensable for dissecting mechanisms of leukocyte guidance and cross-talk in controlled environments. Collectively, these advanced technologies effectively bridge the gap between conventional 2D cultures and animal models, enhancing translational potential while minimizing animal testing. They have been applied to investigate neutrophil swarming, T cell tumor infiltration, and dendritic cell migration. The integration of immune-competent organoids, omics technologies, and machine learning will further improve the precision and predictive value of these *in vitro* systems, driving advances in immunotherapy development and personalized medicine. Recent advances in BBB-on-a-chip models have also enabled the evaluation of S1P_1_ modulators such as siponimod in human-relevant settings (133), while immune-competent organ-on-a-chip platforms-designed to mimic lymphoid tissue microenvironments and cellular cross-talk-offer a promising foundation to investigate mechanisms of Treg recruitment, including chemokine-driven cues such as CCL22 (134).

In summary, advanced *in vitro* models provide powerful and transformative tools to interrogate immune cell chemotaxis with high precision and translational relevance. By bridging the gap between conventional 2D cultures and animal models, they accelerate the discovery of clinically actionable mechanisms. Nonetheless, achieving standardization, scalability, and clinical validation remains essential to fully integrate these platforms into next-generation drug development and precision immunotherapy.

## 5 Emerging technologies and innovative strategies

Emerging studies demonstrate that spatial modeling of the tumor immune microenvironment (TIME) significantly enhances immunotherapy precision. Bagaev et al. identified spatially-defined immune subtypes that predict treatment response and guide combination strategies (135). To better capture the spatial heterogeneity of tumor immunity, the TIME has been classified into three distinct phenotypes: inflamed, immune-excluded, and immune-desert (136). This classification is based on the spatial organization of immune cells, which critically determines therapeutic efficacy. Using high-dimensional imaging, Xiao et al. correlated specific immune cell arrangements with outcomes in triple-negative breast cancer (137). In NSCLC, Patkar et al. developed HistoTME, a deep learning model that predicts TIME composition directly from histology slides, and demonstrated its superiority over traditional biomarkers such as PD-L1 expression (138). Spatial transcriptomics in pancreatic cancer revealed immunosuppressive niches formed by tumor cells, fibroblasts, and dysfunctional T cells. In colorectal cancer, Schürch et al. mapped cellular neighborhoods and found that proximity between antigen-presenting cells and cytotoxic T cells predicted better prognosis (139). Recent integration of spatial and single-cell techniques has enabled construction of a breast cancer immune atlas, demonstrating how cellular spatial positioning and functional states collectively influence treatment outcomes (140). Collectively, these advances underscore spatial immune profiling’s pivotal role in deciphering tumor-immune interactions and shaping immunotherapy strategies, positioning it as a cornerstone for the next generation of precision immuno-oncology.

Beyond spatial analysis, emerging technologies are revolutionizing immune cell migration studies. Intravital microscopy now permits real-time, 3D tracking of immune cell dynamics in living tissues, revealing novel behaviors in health and disease. For instance, a recent study has captured detailed three-dimensional T cell migration trajectories in intestinal mucosa (141). High-throughput CRISPR screening has revolutionized the identification of immune migration regulators. Using pooled sgRNA libraries delivered via AAV or lentiviral vectors in animal models, this approach enables genome-wide gene perturbation. Following *in vivo* selection (e.g., isolating tissue-infiltrating immune cells), next-generation sequencing quantifies sgRNA abundance to reveal migration-modulating genes through enrichment/depletion analysis. Notably, a genome-wide screen in a multiple sclerosis model identified 18 enhancers and 5 suppressors of T cell CNS infiltration, with clinically relevant targets such as α4-integrin, CXCR3, and GRK2 that directly align with mechanisms of approved MS therapies (142). Spatial agent-based models are now being used to simulate chemokine gradients, immune cell interactions, and therapeutic interventions, enabling the prediction of synergistic effects in multi-pathway combination therapies. Mongeon et al. reported that spatial modeling substantially improves immunotherapy outcome predictions (143). Further advances in computational simulations and multi-target prediction models have improved the analysis of immune cell migration dynamics and therapeutic optimization. For instance, spatial agent-based models integrating chemokine gradients, adhesion molecule expression, and cellular communication have been shown to accurately predict the synergistic effects of blocking multiple migratory pathways in combination therapies (142, 144). Similarly, molecular docking studies of natural compounds—such as curcumin, resveratrol, and quercetin-identified interactions with CXCR7, supporting rational drug design efforts (145). More recently, machine learning-driven multi-target prediction platforms have enabled the simultaneous prioritization of candidate molecules acting on chemokines, integrins, and proteases, thereby facilitating the development of broad-spectrum or combination anti-migration agents with improved specificity and reduced toxicity (146).2.20 Building on these computational advances, intelligent drug delivery platforms offer new levels of precision. Environment-responsive nanoparticles that release cargo in response to pH or redox cues within inflammatory microenvironments can minimize off-target effects while fine-tuning immune cell recruitment. Wang et al. and Torchilin et al. reviewed these nanocarrier strategies in detail (147, 148), while more recent work has demonstrated inflammation-responsive biomimetic nanoparticles for targeted therapy (149).

**Figure 3:**
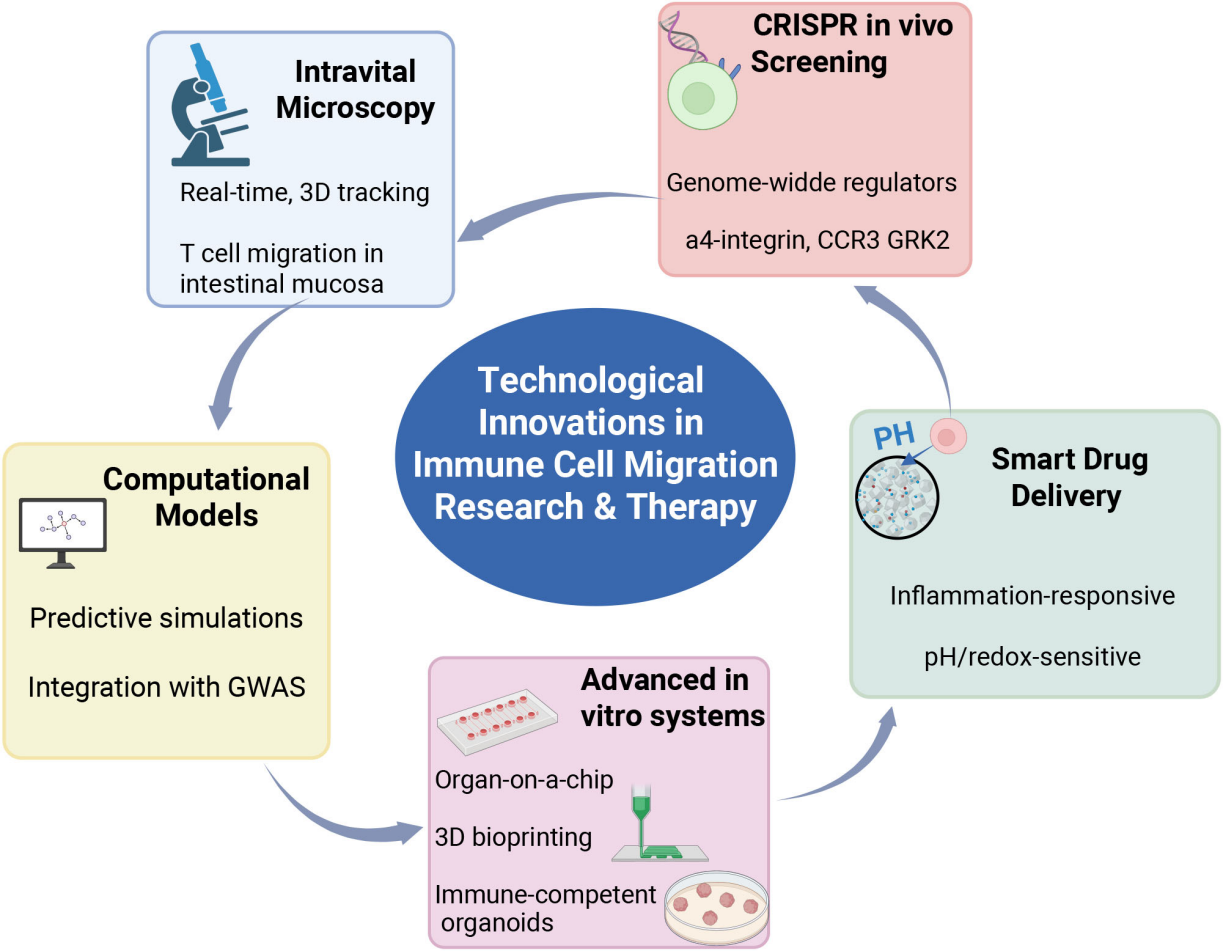
Technological innovations in immune cell migration research and therapy. This figure summarizes recent cutting-edge technologies that have advanced the understanding of immune cell migration mechanisms and related therapeutic strategies. Intravital microscopy enables real-time, three-dimensional tracking of T cell migration. CRISPR-based *in vivo* screening, together with integration with genome-wide association studies (GWAS), facilitates the identification of regulatory factors and predictive simulations. Advanced *in vitro* systems, including organ-on-a-chip platforms, 3D bioprinting, and immune-competent organoids, provide precise models to investigate immune cell trafficking. In parallel, smart drug delivery systems, such as inflammation-responsive and pH/redox-sensitive nanocarriers, are driving translational applications targeting immune cell migration. Potential key regulators, including α4-integrin, CCR3, and GRK2, are also highlighted.

In summary, integrating spatial modeling, advanced imaging, CRISPR screening, and computational simulations has greatly expanded our capacity to dissect immune migration and improve immunotherapy design. However, standardization of spatial profiling and validation of computational predictions in clinical cohorts remain major research gaps that must be addressed before these strategies can be broadly applied in precision oncology (**Figure 3**).

## 6 Future prospects

One promising direction to overcome current limitations in anti-inflammatory therapies is the development of narrow-spectrum migration inhibitors that selectively block the trafficking of disease-relevant immune cell subsets while sparing protective populations critical for immune homeostasis. This selectivity can be achieved through several advanced strategies: (1) nanoparticle-based delivery systems, which preferentially accumulate in inflamed tissues and locally release inhibitors, thereby reducing systemic toxicity and off-target effects; (2) prodrug approaches, in which compounds remain inactive in circulation but are activated by inflammation-associated enzymes-such as matrix metalloproteinases-within diseased microenvironments, enabling spatially restricted action; and (3) targeted protein degradation technologies, including PROTACs (proteolysis-targeting chimeras) and molecular glues, which allow selective elimination of key migration-related proteins (e.g., CCR2, essential for monocyte recruitment), offering precision without broad immunosuppression. Collectively, these precision strategies enhance the therapeutic index of anti-migration treatments and address the enduring challenge of immune selectivity.

**Figure 4:**
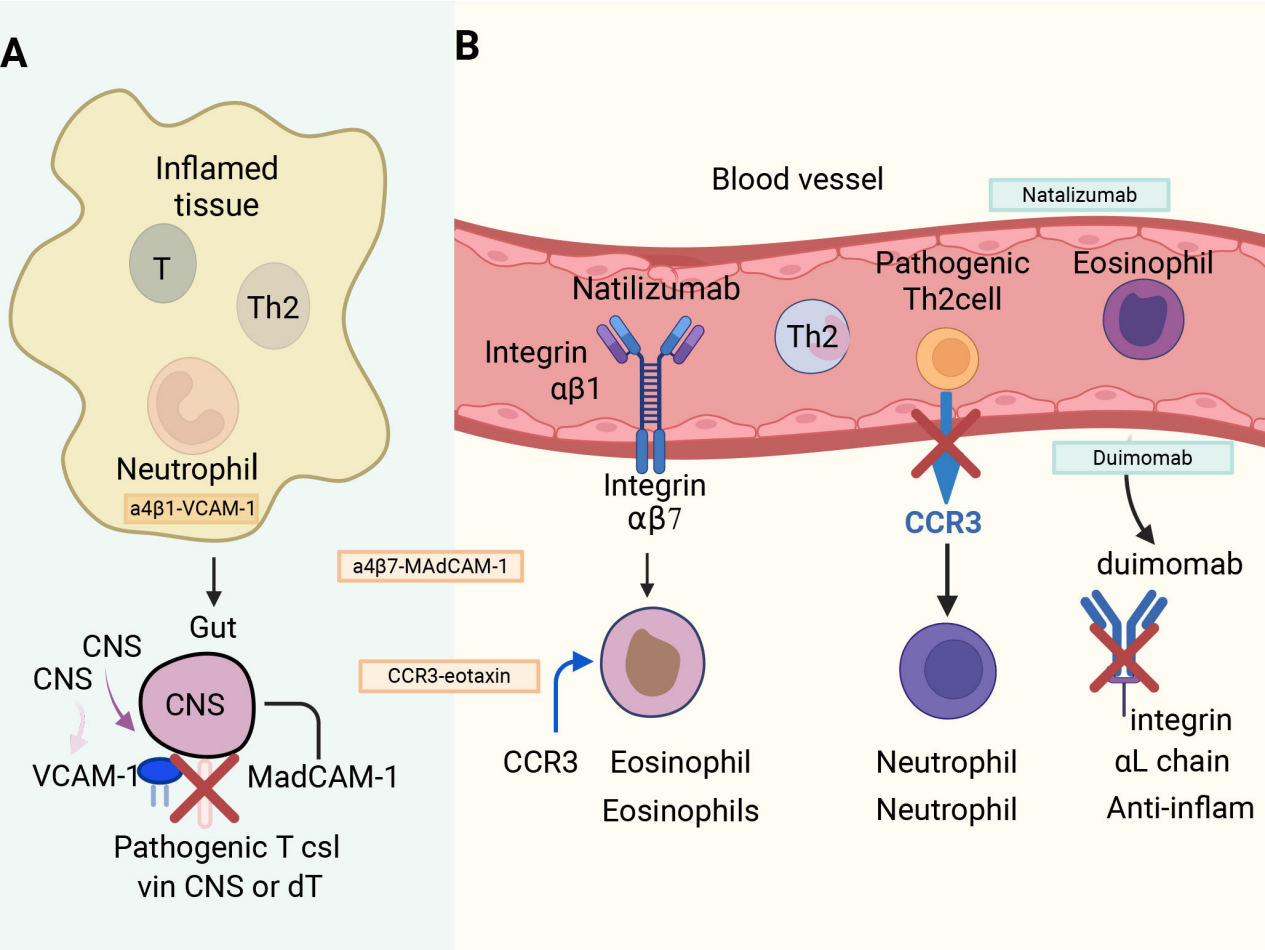
Therapeutic targets of immune cell trafficking. **(A)** Current mechanisms of immune cell migration into inflamed tissues. Pathogenic T cells, Th2 cells, neutrophils, and eosinophils migrate from blood vessels into inflamed tissues via interactions with adhesion molecules and chemokine receptors. Integrin α4β1-VCAM-1 and α4β7-MAdCAM-1 mediate T cell entry into the CNS and gut, respectively. CCR3-eotaxin signaling regulates eosinophil recruitment. **(B)** Approved antibody-based therapies targeting immune cell trafficking. Natalizumab blocks α4 integrin (α4β1/α4β7) to prevent pathogenic T cell and Th2 cell entry into the CNS and gut. Duimomab inhibits the integrin αL chain, reducing neutrophil-mediated inflammation. CCR3 antagonists block eosinophil migration. Colored arrows indicate functional directions: solid arrows (black) = migration/activation pathways; colored arrows = specific chemokine-receptor interactions (e.g., CCR3-eotaxin); red “X” = inhibition by therapeutic antibodies. CNS, central nervous system; VCAM-1, vascular cell adhesion molecule-1; MAdCAM-1, mucosal addressin cell adhesion molecule-1; CCR3, C-C chemokine receptor type 3.

In this review, we have systematically delineated the intricate regulatory mechanisms governing immune cell migration in inflammatory diseases while illuminating the therapeutic potential of modulating these pathways. While substantial progress has been achieved in identifying critical molecular targets and developing precision inhibitors, persistent challenges demand attention-particularly in achieving cell-type specificity, mitigating off-target effects, and bridging the gap between preclinical discovery and clinical translation. Looking forward, emerging technologies such as intravital imaging, CRISPR-based functional genomics, and high-resolution computational modeling are revolutionizing our ability to dissect immune cell behavior *in vivo* and to rationally guide therapy design. Furthermore, innovative therapeutic paradigms-including tissue-specific delivery systems, combinatorial inhibition of multiple pathways, and biomarker-informed personalization-hold great promise in overcoming current therapeutic limitations. As our mechanistic insights continue to expand, these synergistic advancements are poised to catalyze the development of next-generation anti-inflammatory interventions that combine enhanced safety profiles with superior efficacy-precisely disrupting pathogenic immune migration while safeguarding physiological immune function (**Figure 4**).
